# Hemoglobin and cerebral hypoxic vasodilation in humans: Evidence for nitric oxide-dependent and *S*-nitrosothiol mediated signal transduction

**DOI:** 10.1177/0271678X231169579

**Published:** 2023-04-12

**Authors:** Ryan L Hoiland, David B MacLeod, Benjamin S Stacey, Hannah G Caldwell, Connor A Howe, Daniela Nowak-Flück, Jay MJR Carr, Michael M Tymko, Geoff B Coombs, Alexander Patrician, Joshua C Tremblay, Michelle Van Mierlo, Chris Gasho, Mike Stembridge, Mypinder S Sekhon, Damian M Bailey, Philip N Ainslie

**Affiliations:** 1 Department of Anesthesiology, Pharmacology and Therapeutics, Vancouver General Hospital, University of British Columbia, Vancouver, BC, Canada; 2 Department of Cellular and Physiological Sciences, Faculty of Medicine, University of British Columbia, Vancouver, BC, Canada; 3 Centre for Heart, Lung, and Vascular Health, School of Health and Exercise Sciences, Faculty of Health and Social Development, University of British Columbia Okanagan, Kelowna, BC, Canada; 4 International Collaboration on Repair Discoveries, Vancouver, BC, Canada; 5 Human Pharmacology & Physiology Lab, Department of Anesthesiology, Duke University Medical Center, Durham, NC, USA; 6 Neurovascular Research Laboratory, Faculty of Life Sciences and Education, University of South Wales, Pontypridd, UK; 7 Department of Biomechanical Engineering, University of Twente, Enschede, The Netherlands; 8 Department of Medicine, Division of Pulmonary and Critical Care, Loma Linda University School of Medicine, Loma Linda, CA, USA; 9 Cardiff School of Sport and Health Sciences, Cardiff Metropolitan University, Cardiff, UK; 10 Djavad Mowafaghian Centre for Brain Health, Department of Pathology and Laboratory Medicine, Faculty of Medicine, University of British Columbia, Vancouver, BC, Canada; 11 Division of Critical Care Medicine, Department of Medicine, Vancouver General Hospital, University of British Columbia, Vancouver, BC, Canada

**Keywords:** Cerebral blood flow, hemoglobin, hypoxia, nitric oxide, oxygen delivery

## Abstract

Cerebral hypoxic vasodilation is poorly understood in humans, which undermines the development of therapeutics to optimize cerebral oxygen delivery. Across four investigations (total n = 195) we investigated the role of nitric oxide (NO) and hemoglobin-based *S*-nitrosothiol (RSNO) and nitrite ( ${\mathrm{NO}}_{2}^{-}$ ) signaling in the regulation of cerebral hypoxic vasodilation. We conducted hemodilution (n = 10) and NO synthase inhibition experiments (n = 11) as well as hemoglobin oxygen desaturation protocols, wherein we measured cerebral blood flow (CBF), intra-arterial blood pressure, and in subsets of participants trans-cerebral release/uptake of RSNO and ${\mathrm{NO}}_{2}^{-}$ . Higher CBF during hypoxia was associated with greater trans-cerebral RSNO release but not ${\mathrm{NO}}_{2}^{-}$ , while NO synthase inhibition reduced cerebral hypoxic vasodilation. Hemodilution increased the magnitude of cerebral hypoxic vasodilation following acute hemodilution, while in 134 participants tested under normal conditions, hypoxic cerebral vasodilation was inversely correlated to arterial hemoglobin concentration. These studies were replicated in a sample of polycythemic high-altitude native Andeans suffering from excessive erythrocytosis (n = 40), where cerebral hypoxic vasodilation was inversely correlated to hemoglobin concentration, and improved with hemodilution (n = 6). Collectively, our data indicate that cerebral hypoxic vasodilation is partially NO-dependent, associated with trans-cerebral RSNO release, and place hemoglobin-based NO signaling as a central mechanism of cerebral hypoxic vasodilation in humans.

## Introduction

Hypoxic vasodilation is a fundamental physiologic function conserved across mammalian species.^1^ Integral to the maintenance of systemic oxygen (O_2_) delivery, hypoxic vasodilation is requisite to maintain cerebral homeostasis. A high oxygen demand and limited ability for storage of glycolytic substrate underlies the brain’s vulnerability to reductions in oxygen supply. Indeed, complete interruption of cerebral blood flow (CBF), and thus cerebral delivery of oxygen (CDO_2_) precipitates unconsciousness in as little as 4-seconds, with adverse consequences ensuing shortly thereafter.^2^ Clinically, optimizing CDO_2_ remains an elusive target in the setting of acute ischemic brain diseases (e.g. focal ischemic stroke or global hypoxic-ischemic brain injury), while chronic impairments in cerebral perfusion have been associated with increased risk of neurodegenerative disease.^3^ Delineation of the mechanisms regulating cerebral hypoxic vasodilation is necessary to determine future therapies for the optimization of cerebral perfusion.

Nitric oxide (NO) has been demonstrated as a key regulator of cerebral hypoxic vasodilation in animal models.^4,5^ *S*-nitrosohemoglobin, an NO moiety covalently bound to the cysteine 93 residue of the hemoglobin (Hb) β-chain, is now well described to subserve NO-mediated vascular signaling and is a fundamental regulator of hypoxic vasodilation.^6,7^ Allostery dependent, this Hb-based signaling mechanism functions to ‘protect’ NO during circulation^8^ and facilitate export of vasodilatory *S*-nitrosothiols (RSNO) from the erythrocyte during gas exchange at the tissue level.^7–9^ Indeed, pre-clinical studies have established that RSNO release from hemoglobin during circulatory transit across a vascular bed occurs commensurate to oxygen demand.^8,9^ Hypoxic vasodilation may also be transduced through a nitrite ( ${\mathrm{NO}}_{2}^{-}$ ) reductase activity of deoxyhemoglobin,^10^ which has also been demonstrated to contribute to vascular reactivity to hypoxia.^11^ Yet, limited work in humans has addressed NO-dependent regulation of cerebral hypoxic vasodilation, wherein methodological limitations of previous work have led to inconsistent and irreconcilable findings.^12,13^

Given the apparent multi-dimensional regulation of cerebral hypoxic vasodilation in humans, we conducted a series of four inter-related studies with the central aim of determining the regulation of cerebral hypoxic vasodilation by Hb and NO. We first aimed to determine the roles that RSNO and ${\mathrm{NO}}_{2}^{-}$ play in cerebral hypoxic vasodilation, and hypothesized *a priori* that the magnitude of cerebral hypoxic vasodilation would be associated with trans-cerebral RSNO release, thus providing evidence for NO-dependency. Second, we aimed to determine the impact of NO synthase inhibition on cerebral hypoxic vasodilation and hypothesized *a priori* that cerebral hypoxic vasodilation would be reduced following NO synthase inhibition. To assess these first two hypotheses, we conducted invasive studies in healthy humans to assess trans-cerebral NO signaling in response to matched levels of hypoxia elicited by hypoxemia (reduced arterial oxygen saturation, SaO_2_) and experimental isovolumic hemodilution (to reduce [Hb]) as well as the influence of pharmacologic NO synthase inhibition on cerebral hypoxic vasodilation. Third, given the influence of Hb on NO bioavailability and signal transduction, we aimed to determine the relationship between [Hb] and cerebral hypoxic vasodilation and hypothesized *a priori* that a lower [Hb] would be associated with a greater cerebral hypoxic vasodilation. To assess our third hypothesis, we measured [Hb] and the magnitude of cerebral hypoxic vasodilation in a cohort of >100 individuals. Finally, we aimed to determine the influence of an elevated [Hb] in patients with excessive erythrocytosis on cerebral hypoxic vasodilation. In keeping with investigations 1–3, we hypothesized *a priori* that a higher [Hb] would be associated with a lesser magnitude of cerebral hypoxic vasodilation, while reducing [Hb] via hemodilution would increase cerebral hypoxic vasodilation. To assess our fourth hypothesis we investigated the relationship between arterial [Hb] and cerebral hypoxic vasodilation in polycythemic high-altitude native Andeans with excessive erythrocytosis prior to and following experimental isovolumic hemodilution. Our results broadly indicate that cerebral hypoxic vasodilation is NO-dependent and RSNO-mediated, whereby arterial [Hb] is inversely related to the magnitude of cerebral hypoxic vasodilation in response to graded hypoxemia.

## Methods

We conducted four separate investigations wherein we examined: 1) the association between RSNOs and cerebral hypoxic vasodilation, 2) the NO-dependence of cerebral hypoxic vasodilation, 3) the association between [Hb] and cerebral hypoxic vasodilation, and 4) cerebral hypoxic vasodilation in high-altitude native Andeans with and without excessive erythrocytosis. This study adhered to the standards outlined in the Declaration of Helsinki (except registry in a database) and the Canadian Tri-council Policy Statement for Integrity in Research. All protocols and retrospective analyses were approved by the University of British Columbia’s Clinical Research Ethics Board (H16-01028; H18-01755; H18-01404; H18-01764), Duke University Institutional Review Board (Protocol: 00091879), and the Universidad Peruana Cayetano Heredia Comité de Ética (no. 101686).^14^ All participants read an in-depth study information form, spoke with an investigator, and provided written informed consent prior to participation (in their native language). Some aspects of investigations 1 and 2 have been reported previously (e.g. blood gas data),^15,16^ however, the main outcomes reported herein and the hypotheses addressed hold no overlap.

### Investigation 1 – Isovolumic hemodilution

Ten young, healthy males were recruited to participate (Age: 29 ± 7 years; body mass index, BMI: 23 ± 2 kg · m^−2^). Following instrumentation with radial arterial, jugular venous (see Supplemental Figure 1), and ante-cubital venous catheters, participants had ≥20 minutes to rest while the remaining experimental set-up for monitoring minute ventilation (V̇_E_), the partial pressures of end-tidal carbon dioxide (P_ET_CO_2_) and oxygen (P_ET_O_2_), heart rate (HR), radial artery mean arterial pressure (MAP), jugular venous blood pressure (JVBP), and cerebral perfusion pressure (CPP, i.e. MAP – JVBP) (see Supplemental Methods). Moreover, during each measurement period, vertebral (VA) and internal carotid artery (ICA) blood flow was quantified to determine CBF in conjunction with blood gas analysis (see Supplemental Methods). For each experimental manipulation, end-tidal forcing was utilized to control arterial blood gases. After 5-minutes of eucapnic and normoxic baseline measurements, each participant completed an isocapnic hypoxemia test with targeted arterial oxygen saturation (SaO_2_) values of 90, 85, and 75%. Isovolumic hemodilution was then performed, where 10% of whole blood volume was removed and replaced with an equal volume of 5% human serum albumin (Alburex® 5%, Canadian Blood Services) in two successive stages, resulting in a total removal and replacement of 20% of whole blood volume. Baseline (eucapnic normoxia) measures were acquired following the first stage of 10% blood removal/replacement, while a second isocapnic hypoxemia trial was conducted following the second stage of hemodilution (see Supplemental Methods). At each baseline and during each hypoxemia test, V̇_E_, P_ET_CO_2_, P_ET_O_2_, HR, MAP, JVBP, VA, ICA, and blood sampling measures were performed, with data averaged over the last minute of each stage.

### Investigation 2 – Nitric oxide synthase inhibition

Eleven young, healthy males were recruited to participate (Age: 25 ± 5 years; BMI: 24 ± 3 kg/m^2^). Following instrumentation (consistent with Investigation 1), either a control (volume matched saline, 0.9% NaCl), NO synthase inhibitor (N^G^-monomethyl-L-arginine, L-NMMA) or α_1_-adrenoceptor agonist (phenylephrine, n = 5 subset) infusion was initiated (singled-blinded, counter-balanced design). The L-NMMA infusion was initiated with a 5 mg/kg bolus delivered over 5 min followed by a 50 μg/kg/min maintenance dose throughout the protocol until completion. Phenylephrine was infused at 0.1 to 0.6 μg/kg/min to serve as an equipotent vasoconstrictor control for the L-NMMA trial.^12^ During each drug infusion we utilized end-tidal forcing to conduct an isocapnic hypoxemia test with a baseline period (eucapnic normoxia) followed by two hypoxic stages lasting 5-minutes each where SaO_2_ was targeted to 85% and 75%. At baseline and each stage of hypoxemia, V̇_E_, P_ET_CO_2_, P_ET_O_2_, HR, MAP, JVBP, MCA, PCA, VA, ICA, and blood sampling measures were performed.

#### Investigation 3 – Baseline hemoglobin concentration and cerebral hypoxic vasodilation

We conducted a retrospective analysis of nine experiments wherein participants were instrumented with a radial artery and jugular venous catheter and completed an isocapnic hypoxemia test. From these nine separate experiments conducted between 2004 and 2016, we assessed all data files (n = 199) with 134 participants included in our final analysis (79 male, 55 female; Age: 26 ± 6 years; BMI: 24 ± 3 kg/m^2^) (see Supplemental Methods for exclusion criteria). Concurrent arterial and jugular venous blood samples were acquired serially at each stage of hypoxemia, wherein hypoxia was induced utilizing a sequential gas delivery system (RespirAct®, Thornhill Medical, Toronto, Canada).^17^ Percent changes in CBF were calculated based upon the Fick principle under the assumption that cerebral metabolism of oxygen remains constant during the level of hypoxemia employed in this study^18^ (i.e., CBF ∝ ${\text{a-vDO}}_{2}^{-1}$ ; see Supplemental Methods).

#### Investigation 4 – cerebral hypoxic vasodilation in the setting of lifelong hypoxia

In order to isolate the role of elevations in [Hb] on cerebral hypoxic vasodilation, high-altitude native Andeans with (n = 15; all male; Age: 39 ± 15 years; BMI: 25 ± 3 kg/m^2^) and without (n = 25; 2 female; Age: 29 ± 12 years; BMI: 24 ± 3 kg/m^2^) excessive erythrocytosis (EE+/EE−) were recruited to participate in the Cerro de Pasco region of Peru (4,300 m above sea-level). Venous blood was collected for the measurement of Hct and [Hb], and CBF was measured with duplex ultrasound. Utilizing an end-tidal forcing system, an isocapnic hypoxemia test was performed in 15 participants, wherein P_ET_O_2_ was targeted to 100 mmHg to restore SaO_2_ to normal (i.e. 98%), and then reduced to a P_ET_O_2_ of 50 and 40 mmHg, with each stage lasting 5-minutes following the attainment of steady-state. At each stage, V̇_E_, P_ET_CO_2_, P_ET_O_2_, HR, MAP, VA, and ICA measures were made (see Supplemental Methods). Further, a subset of six EE+ males were instrumented with a radial artery catheter and completed an isovolumic hemodilution protocol (Age: 44 ± 19 years; BMI: 25 ± 3 kg/m^2^). Therein, 25% of blood volume was removed and replaced with human serum albumin (5%) as described in Investigation 1. Prior to and following hemodilution these participants underwent an isocapnic hypoxemia test as described above.

#### Statistical analyses

For all statistical tests, significance was set at an alpha level of α = 0.05. Data were assessed for normality using a Shapiro-Wilk test. Normal data are reported as mean ± standard deviation. When data significantly differed from a normal distribution, non-parametric analyses were employed and data are presented as median [interquartile range]. Across all investigations, pre vs. post intervention or between group comparisons were performed using two-tailed t-tests or non-parametric equivalents (Mann Whitney U test or Wilcoxon Signed Ranks test). Responses during hypoxemia tests prior to and following an intervention were made using linear mixed models or two-way ANOVAs. Finally, correlational analyses were conducted with Pearson r correlations or repeated measures correlations.^19^ See Supplemental Methods.

## Results

### Investigation 1 – Cerebral hypoxic vasodilation is associated with trans-cerebral S-nitrosothiol release

**Figure 1.**
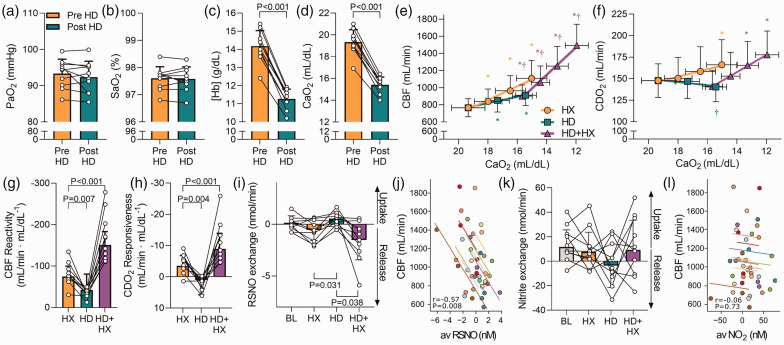
Cerebral hypoxic vasodilation is associated with trans-cerebral release of *S*-nitrosothiols. Panels (a–d) depict PaO_2_, SaO_2_, [Hb], and CaO_2_ at baseline pre- and post-hemodilution, respectively. Panel e depicts CBF during each stage across all trials. CBF increased at each stage of hypoxemia (HX), hemodilution (HD), and combined hemodilution and hypoxemia (HD + HX) compared to their respective eucapnic and normoxic baselines (P < 0.05 for all). Further, CBF was higher at each stage of combined HD + HX compared to each SaO_2_ matched stage of HX (P < 0.05 for all). Panel f depicts CDO_2_ during each stage across all trials. CDO_2_ was increased from baseline (148 ± 19 mL/min) at the third HX stage (166 ± 29 mL/min; P = 0.002), while CDO_2_ was lower at the third HD stage compared to the CaO_2_ matched HX stage (140 ± 17 mL/min; P = 0.001). Similarly, following hemodilution, hypoxemia increased CDO_2_ from the post-hemodilution baseline (141 ± 19 mL/min) at the second (166 ± 28 mL/min; P < 0.001) and third stage of hypoxemia (178 ± 28 mL/min; P < 0.001). Panels g and h depict the individual CBF and CDO_2_ slope responses to reductions in CaO_2_ across each trial, respectively. Panels i and k depict the trans-cerebral exchange of *S*-nitrosothiols (RSNO) and ${\mathrm{NO}}_{2}^{-}$ at baseline and the final stage of the HX, HD, and HD + HX trials, respectively and Panels j and l depict the repeated measures correlation analyses between CBF and the arterial-venous differences of RSNO and ${\mathrm{NO}}_{2}^{-}$ , respectively. Different color symbols and regression lines represent different participants. N=10 for all comparisons. An asterisk (*) indicates a significant difference from baseline within a stage, while a dagger symbol (†) indicates a difference between trials (compared to HX) for a matched CaO_2_ (i.e. HX vs HD comparisons) or SaO_2_ (i.e. HX vs HD + HX comparisons). Significance was set at P < 0.05. Comparisons without an associated p-value or symbol were not significant.

We utilized our isovolumic hemodilution protocol to match changes in CaO_2_ between hypoxemic and anemic stages of hypoxia. Neither PaO_2_ (93.3 ± 4.0 vs. 92.4 ± 4.4 mmHg; P = 0.27) nor SaO_2_ (97.6 ± 0.4 vs. 97.6 ± 0.6%; P = 0.75) were altered pre-to post hemodilution (Figure 1(a) and (b)). However, the reduction in [Hb] from 14.2 ± 0.9 to 11.3 ± 0.5 mL/dL (P < 0.001) led to a 20 ± 2% reduction in CaO_2_ (19.3 ± 1.1 vs. 15.4 ± 0.7 mL/dL; P < 0.001) (Figure 1(c) and (d)). There was a 19 ± 7% increase in CBF following hemodilution (765 ± 105 vs. 912 ± 120 mL/min; P < 0.001).

The CBF and CDO_2_ responses during the three hypoxic tests are depicted in Figure 1(e) and (f). The CBF reactivity to hypoxemia prior to hemodilution was −74.8 ± 25.0 mL/min · mL/dL^−1^. Reactivity to anemic hypoxia (the hemodilution protocol) was −42.1 ± 38.4 mL/min^−1^ · mL/dL^−1^, ∼45% lower than that during hypoxemia (P = 0.007). Reactivity to hypoxemia following hemodilution was ∼100% greater than hypoxemia prior to isovolumic hemodilution (−150.6 ± 32.4 mL/min · mL/dL^−1^; P < 0.001; Figure 1(g)). Inclusion of CPP as a covariate did not alter these differences. The slope response of CDO_2_ (i.e. CDO_2_ reactivity) to hypoxemia prior to hemodilution was −3.5 ± 3.3 mL/min · mL/dL^−1^. Conversely, hemodilution caused a progressive decrease in CDO_2_ in concert with CaO_2_ (CDO_2_ reactivity, 1.0 ± 4.8 mL/min · mL/dL^−1^; P = 0.004). Indeed, CDO_2_ was lower during anemic hypoxia (140.3 ± 17.0 mL/dL) compared to hypoxemia (166.1 ± 29.2 mL/dL; P = 0.001; Figure 1(e)) despite matched CaO_2_. Finally, CDO_2_ reactivity to hypoxemia following hemodilution was ∼150% greater than pre hemodilution (−8.9 ± 5.0 mL/min · mL/dL^−1^; P < 0.001; Figure 1(h)).

Cerebral release of RSNO was greater during both the final stage of hypoxemia pre-hemodilution (−0.54 ± 1.03 nmol/min; P = 0.031) and final stage of hypoxemia post-hemodilution (−1.51 ± 1.92 nmol/min; P = 0.038) compared to the final stage of hemodilution (0.54 ± 0.66 nmol/min) (Figure 1(i)). This was reflected in a correlation^19^ between the magnitude of the cerebral a-v RSNO difference and CBF (r = −0.57; P = 0.0080; Figure 1(j)). No differences in the cerebral net exchange of ${\mathrm{NO}}_{2}^{-}$ were observed (P = 0.30; Figure 1(k)) and there was no relationship between the a-v ${\mathrm{NO}}_{2}^{-}$ difference and CBF (r = −0.06; P = 0.73; Figure 1(l)).

### Investigation 2 – Nitric oxide synthase inhibition reduces cerebral hypoxic vasodilation

**Figure 2.**
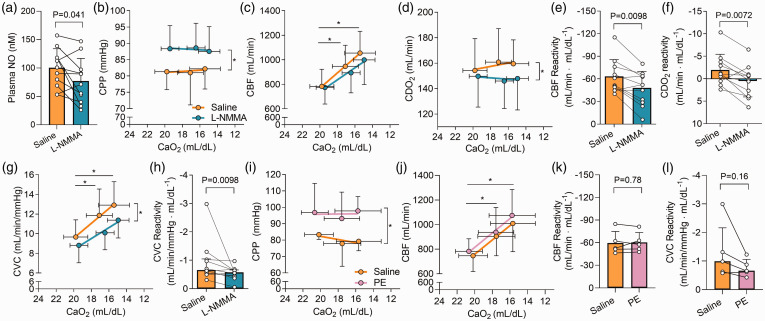
Nitric oxide synthase inhibition reduces cerebral hypoxic vasodilation in response to progressive hypoxemia. Panel a depicts plasma NO ( ${\mathrm{NO}}_{2}^{-}$ + RSNO) at baseline during saline (orange) and L-NMMA infusion (cyan). Panels b–d depict CPP, CBF, and CDO_2_ in response to hypoxemia during the saline and L-NMMA infusion. Panels e and f depict the magnitude of cerebral hypoxic vasodilation (i.e. CBF reactivity) and CDO_2_ response to hypoxemia during the saline and L-NMMA infusions. Panels g and h depict the changes in CVC with hypoxemia and resulting CVC reactivity slopes, respectively, during saline and L-NMMA infusion. To take into account the known vasopressor influence of L-NMMA, we ran an equipotent vasoconstrictor control trial with phenylephrine (PE) in 5 participants. Panels i and j depict cerebral perfusion pressure (CPP) and CBF in response to hypoxemia during both saline (orange) and phenylephrine trials (pink) and Panels k and l depict the CBF and CVC reactivity, respectively, during saline and phenylephrine infusion. Bar graphs represent mean ± standard deviation with individual data overlaid. Line plots represent mean ± standard deviation. N = 11 for statistical testing between saline and L-NMMA, while n = 5 for statistical testing between saline and phenylephrine. For panels b, c, d, g, i, & j, an asterisk (*) indicates a significant main effect of either stage of hypoxemia or drug infusion (P < 0.05). Comparisons without an associated p-value or symbol were not significant.

Arterial plasma total NO ( ${\mathrm{NO}}_{2}^{-}$ + RSNO) was 23% lower in the L-NMMA trial compared to saline (77.1 ± 39.9 vs. 100.4 ± 33.8 nM; P = 0.041; Figure 2(a)). We effectively matched PaO_2_ (P = 0.14) and CaO_2_ (P = 0.43) between the saline and L-NMMA hypoxemia tests while maintaining isocapnia (P = 0.45). Given the vasoconstrictor influence of NO synthase inhibition, CPP was elevated during L-NMMA infusion compared to saline (P < 0.001; Figure 2(b)). Isocapnic hypoxemia increased CBF from baseline (eucapnic normoxia) for both trials (main effect, P < 0.001; Figure 2(c)), yet, CDO_2_ was lower during L-NMMA infusion compared to saline (P = 0.002; Figure 2(d)). The CBF reactivity was 24% lower during L-NMMA infusion compared to saline infusion (−63.3 ± 22.3 vs. −48.2 ± 21.9 mL/min · mL/dL^−1^; P = 0.0098; Figure 2(e)). Moreover, CDO_2_ reactivity was 125% lower during L-NMMA infusion than with saline (0.49 ± 3.54 vs. −1.96 ± 3.47 mL/min · mL/dL^−1^; P = 0.0072; Figure 2(f)). As CPP was higher with L-NMMA, and to account for individual participant differences in CPP, we calculated cerebrovascular conductance (CVC) (Figure 2(g)), and the slope response of CVC to hypoxemia was 41% lower during L-NMMA infusion compared to saline infusion (−0.94 ± 0.74 vs. −0.56 ± 0.23 mL/min/mmHg · mL/dL^−1^; P = 0.0098; Figure 2(h)).

We assessed the influence of phenylephrine infusion, a vasoconstrictor control, on cerebral hypoxic vasodilation compared to saline. Phenylephrine led to a higher CPP compared to saline (P < 0.001; Figure 2(i)). However, while CBF was increased with hypoxemia (P < 0.001), there was no difference in CBF between saline and phenylephrine trials (P = 0.147; Figure 2(j)). Ultimately, neither CBF reactivity (−59.6 ± 15.3 vs. −60.4 ± 13.1 mL/min · mL/dL^−1^; P = 0.78; Figure 2(k)) or CVC reactivity (−0.99 [−2.16–−0.61] vs. −0.66 [−1.06–−0.53] mL/min/mmHg · mL/dL^−1^; P = 0.125; Figure 2(l)) were different between saline and phenylephrine trials.

### Investigation 3 – Lower hemoglobin concentration is associated with increased cerebral hypoxic vasodilation

**Figure 3.**
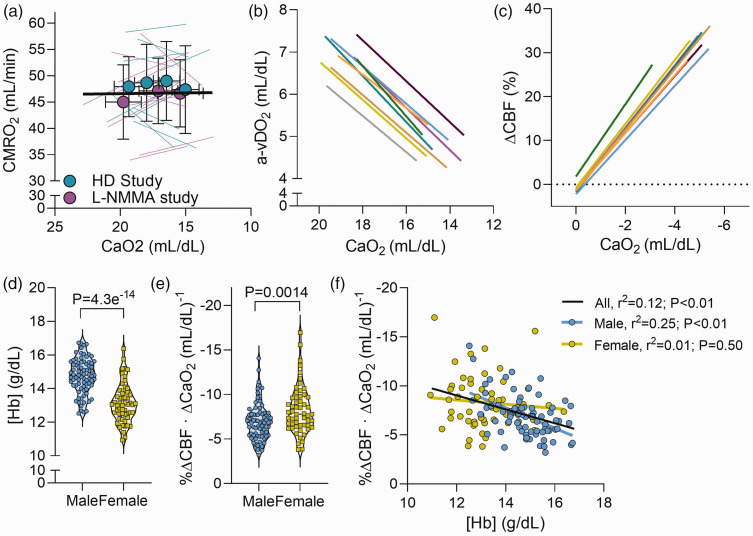
Association between hemoglobin concentration and cerebral hypoxic vasodilation. Panel a depicts the cerebral metabolic rate of oxygen (CMRO_2_) in the 21 participants combined from the hemodilution study (HD Study; cyan) and the nitric oxide synthase inhibition study (L-NMMA Study; magenta). Mean ± standard deviation is presented for each study, overlaid on individual participant responses. The black line represents the overall linear regression for the entire n = 21 sample (linear mixed model analysis). Panel b depicts the relationship between CaO_2_ and the a-vDO_2_ during progressive hypoxemia for each study included in this analysis (a different colour indicates a different study). These data are collated from nine separate studies conducted by our group over the last two decades (see Supplemental Methods). As per the Fick Principle (CMRO_2_ = a-vDO_2_ × CBF), in the setting of a constant metabolism, CBF is inversely proportional to a-vDO_2_ (a-vDO_2_ ∝ 1/CBF). Thus, the percent change in CBF can be determined, as depicted in Panel c. Panel d and e depict [Hb] and the magnitude change in CBF change per unit reduction in CaO_2_ (i.e. magnitude of cerebral hypoxic vasodilation), respectively for both males (blue symbols; n = 79) and females (yellow symbols; n = 55). The relationship between [Hb] and the magnitude of cerebral hypoxic vasodilation is depicted in Panel f. The black line represents the regression for all individuals, while the blue and yellow regressions are specific to the male and female participants, respectively. N=134. Comparisons without an associated p-value or symbol were not significant.

In Investigation 1, the CBF response to hypoxemia following hemodilution (i.e. during concurrent anemia) was greater than pre-hemodilution, indicating a modulatory role of [Hb] in the regulation of cerebral hypoxic vasodilation. To confirm this, we assessed the potential relationship between [Hb] and cerebral hypoxic vasodilation across a larger cohort of individuals. As we have previously demonstrated the stability of CMRO_2_ during equivalent hypoxemia,^18^ and herein confirm in 23 participants (from studies 1 & 2; Figure 3(a)) that CMRO_2_ is unaltered with hypoxemia (P = 0.892), we are able to infer percent changes in CBF from changes in the a-vDO_2_ (see Supplemental Methods). Therefore, in 134 individuals (79 male, 55 female; see Supplemental Table 2 for details) we retrospectively analyzed arterial and jugular venous blood gas data to calculate a-vDO_2_ (Figure 3(b)) and percent changes in CBF during hypoxemia (Figure 3(c)). Males had a higher [Hb] (14.8 ± 1.0 g/dL) compared to females (13.1 ± 1.2 g/dL; P = 4.3e^−14^; Figure 3(d)), but lower cerebral hypoxic vasodilation (−6.9 ± 2.1 vs. −8.3 ± 2.7%ΔCBF · mL/dL^−1^; P = 0.0014; Figure 3(e)). Across all individuals we observed an inverse relationship between cerebral hypoxic vasodilation and [Hb] (r^2^ = 0.12; P < 0.01; Figure 3(f)). This relationship remained evident when males were assessed independently (r^2^ = 0.25; P < 0.01; Figure 3(f)); however, it was absent in the female participants (r^2^ = 0.01; P = 0.50; Figure 3(f)).

### Investigation 4 – Reductions in hemoglobin concentration increase cerebral hypoxic vasodilation in patients with excessive erythrocytosis

**Figure 4.**
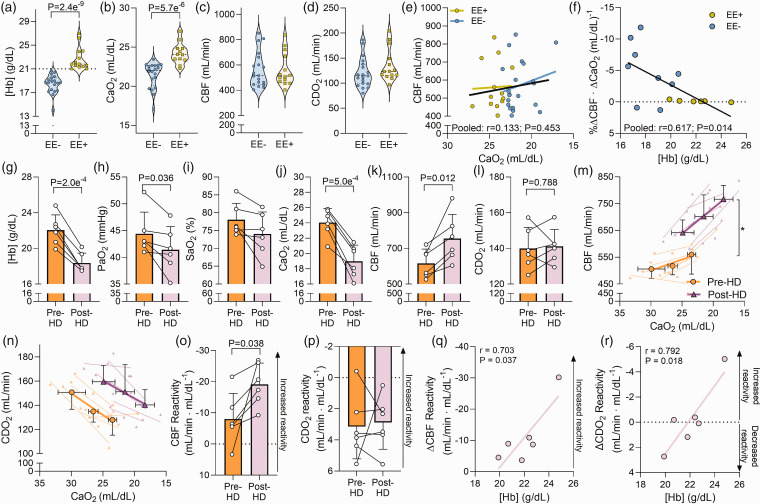
Isovolumic hemodilution increases cerebral hypoxic vasodilation in high-altitude Andeans with excessive erythrocytosis. Panels a–d depict violin plots of hemoglobin concentration [Hb], arterial oxygen content (CaO_2_), cerebral blood flow (CBF), and cerebral delivery of oxygen (CDO_2_) for high-altitude native Andeans without (EE−, n = 19) and with (EE+, n = 14) excessive erythrocytosis. Panel e depicts the relationship between CaO_2_ and CBF under resting conditions (r = 0.133; P = 0.45), while Panel f depicts the relationship between [Hb] and CBF reactivity in a subset of the Andean participants with (n = 6) and without EE (n = 9). Panels g to m depict bar graphs (mean ± standard deviation) with individual data overlaid for [Hb], the partial pressure of arterial oxygen (PaO_2_), arterial oxygen saturation (SaO_2_), CaO_2_, CBF, and CDO_2_, respectively, prior to and following isovolumic hemodilution (HD) in the EE+ group (n = 6). Panels m and n display CBF and CDO_2_ during graded hypoxemia (reduced CaO_2_) prior to and following isovolumic hemodilution, respectively and Panels o and p depicts the CBF and CDO_2_ reactivity, respectively, while Panels q and r depict the relationship between the change in CBF and CDO_2_ reactivity following hemodilution with the pre-hemodilution [Hb]. An asterisk (*) indicates a significant main effect of isovolumic hemodilution (P < 0.05). Comparisons without an associated p-value or symbol were not significant.

To translate our findings from Investigations 1–3 to a clinical population we assessed the potential relationship between [Hb] and cerebral hypoxic vasodilation in high-altitude native Andeans with and without excessive erythrocytosis.^20^ The EE+ participants had a [Hb] of 21.8 [21.4–23.9] g/dL while the EE- participants had a [Hb] of 18.7 [17.3–19.5] g/dL (P = 2.4e^−9^; Figure 4(a)), which corresponded to a higher CaO_2_ (P = 5.7e^−6^; Figure 4(b)). Neither CBF (516 [445–661] vs. 512 [465–682] mL/min; P = 0.93; Figure 4(c)) nor CDO_2_ (115 [99–138] vs. 123 [116–157] mL/min; P = 0.11; Figure 4(d)) differed between the EE− and EE+ groups. There was no correlation between CaO_2_ and CBF at rest (r = 0.133; P = 0.45; Figure 4(e)). However, in agreement with our observations in Investigation 3 (Figure 3(f)), we observed an inverse relationship between arterial [Hb] and CBF reactivity (r = −0.617; P = 0.014; Figure 4(f)).

To assess the influence of reducing [Hb] on CBF reactivity, 6 of the EE+ patients underwent isovolumic hemodilution. This led to a reduction in [Hb] (Figure 4(g)) and CaO_2_ (Figure 4(j)), while SaO_2_ was unaltered (Figure 4(i)) despite a nominal reduction in PaO_2_ (Figure 4(h)). Isovolumic hemodilution was associated with a 22 ± 13% increase in CBF (616.1 ± 79.9 vs. 755.3 ± 135.2 mL/min; P = 0.012; Figure 4(k)) whereas CDO_2_ did not change (Figure 4(l)). In response to hypoxemia, CBF was increased prior to and following isovolumic hemodilution (main effect, P = 0.005; Figure 4(m)) while CDO_2_ decreased (main effect, P = 0.001; Figure 4(n)) both before and after hemodilution. However, comparison of reactivity slopes demonstrated that isovolumic hemodilution increased CBF reactivity by ∼140% (−8.0 ± 8.2 vs. −19.2 ± 6.8 mL/min · mL/dL^−1^; P = 0.038; Figure 4(o)), despite an unaltered CDO_2_ reactivity (P = 0.812; Figure 4(p)). Individual changes in CBF and CDO_2_ reactivity were correlated to pre-hemodilution [Hb], whereby a higher [Hb] prior to hemodilution was associated with a greater improvement in CBF and CDO_2_ reactivity following isovolumic hemodilution (Figure 4(q) and (r)).

## Discussion

Our investigations demonstrate that cerebral hypoxic vasodilation is nitric oxide-dependent, associated with trans-cerebral release of *S*-nitrosothiols, and inversely related in magnitude to arterial hemoglobin concentration. Specifically, we demonstrated that: 1) greater trans-cerebral release of *S*-nitrosothiols during hypoxia is associated greater cerebral blood flow; 2) cerebral hypoxic vasodilation is reduced by 25% following nitric oxide synthase inhibition; 3) the magnitude of cerebral hypoxic vasodilation is inversely correlated to arterial hemoglobin concentration; 4) reducing arterial hemoglobin concentration in the setting of excessive erythrocytosis increases cerebrovascular reactivity to hypoxia. Collectively, our integrated approach implicates nitric oxide as a primary regulator of cerebral hypoxic vasodilation in humans.

**Figure 5.**
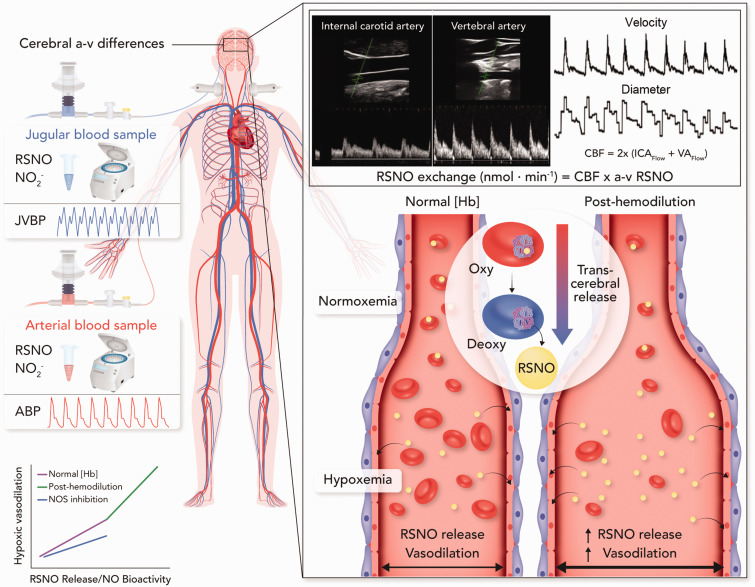
Assessing trans-cerebral exchange kinetics in humans. This schematic depicts our experimental model for assessing the trans-cerebral exchange of signaling molecules (e.g. nitric oxide) in humans. Cerebral arterial-to-venous (a-v) differences were quantified using concurrent arterial and jugular bulb blood sampling. Coupling of the a-v difference of *S*-nitrosothiols (RSNOs) and cerebral blood flow (CBF) measures acquired with duplex ultrasound allows for the quantification of the molar exchange of RSNOs during cerebral circulatory transit. This model of simultaneous arterial and jugular bulb blood sampling allows for experimental isolation of the cerebrovascular bed during a systemic stressor such as arterial hypoxemia or hemodilution and a more specific assessment of the signaling pathways involved in cerebrovascular control. In the present study we investigated the trans-cerebral exchange kinetics of RSNOs and ${\mathrm{NO}}_{2}^{-}$ during arterial hypoxemia, hemodilution, and hypoxemia post-hemodilution. As RSNOs are released from the red blood cell following the allosteric shift from the relaxed (oxygenated) to tense (deoxygenated) conformation, an increase in RSNOs in the venous circulation and thus greater a-v difference is taken to indicate greater release from red blood cells during cerebral circulatory transit. The increase in CBF during arterial hypoxemia in participants with a lower [Hb] as well as those tested post-hemodilution (right side vessel) was greater than in participants with a normal [Hb] and those tested prior to hemodilution (left side vessel). A greater CBF response (i.e. greater cerebral hypoxic vasodilation) was associated with greater trans-cerebral release of RSNOs. The CBF response to hypoxemia was blunted following nitric oxide synthase blockade (bottom left panel). ABP, arterial blood pressure; JVBP, jugular venous blood pressure; NOS, nitric oxide synthase.

To what extent NO contributes to cerebral hypoxic vasodilation in humans has remained equivocal. Albeit limited evidence, prior studies employing an NO synthase blockade have demonstrated reductions^13^ or no impact^12^ on cerebral hypoxic vasodilation, potentially due in part to technical limitations of measuring intra-cranial blood velocity and not flow. We provide clear evidence that NO synthase inhibition reduced cerebral hypoxic vasodilation (Figure 2). As the 23% reduction in plasma total NO with the L-NMMA infusion is not indicative of complete NOS inhibition, the observed 24% reduction in cerebral hypoxic vasodilation with L-NMMA likely underestimates the true magnitude by which NO contributes to cerebral hypoxic vasodilation in humans. This observation is consistent with our hemodilution study where we used differing hypoxic interventions of hypoxemia and hemodilution to elicit CaO_2_ matched levels of hypoxia with differing trans-cerebral RSNO release. We conducted an additional hypoxic test in the setting of acute anemia (i.e. combined hemodilution and hypoxemia) to investigate the influence of reductions in [Hb] on cerebral hypoxic vasodilation and trans-cerebral RSNO release. During these studies we simultaneously collected arterial and jugular bulb blood samples to quantify the arterial-to-venous differences of NO species (RSNO & ${\mathrm{NO}}_{2}^{-}$ ) as well as Duplex ultrasound measures to quantify CBF and subsequently calculate the trans-cerebral exchange of RSNO and ${\mathrm{NO}}_{2}^{-}$ (Figure 5). Across these conditions we observed that the largest increases in CBF (during hypoxemia and combined hemodilution and hypoxemia) were associated with the greatest RSNO release, while the lowest increase in CBF (during hemodilution) was associated with no net trans-cerebral exchange of RSNO (Figure 1). Indeed, as is the case in pre-clinical studies where hemodilution does not increase cyclic guanosine monophosphate (which is a secondary messenger downstream of NO signaling),^21^ the reduction in reactivity observed here was linked to a reduction in RSNO-mediated signaling.

The observation that the magnitude of cerebral hypoxic vasodilation was associated with the magnitude of trans-cerebral RSNO release is in agreement with work by Stamler’s group.^7^ SNO-Hb-mediated hypoxic vasodilation has been demonstrated in pre-clinical *ex vivo*^22^ and *in vivo* models,^8,9^ and verified with genetic knockouts.^1,23^ In humans, however, we saw no such relationship between trans-cerebral exchange of ${\mathrm{NO}}_{2}^{-}$ and cerebral hypoxic vasodilation. An important limitation to consider is that we did not conduct analysis of red blood cell ${\mathrm{NO}}_{2}^{-}$ or iron nitrosylhemoglobin (HbNO), and cannot directly account for reduction of ${\mathrm{NO}}_{2}^{-}$ within the red blood cell (i.e. a trans-cerebral reduction in red blood cell ${\mathrm{NO}}_{2}^{-}$ ). Nonetheless, this lack of relationship with trans-cerebral ${\mathrm{NO}}_{2}^{-}$ release and CBF is relevant given hemoglobin-based ${\mathrm{NO}}_{2}^{-}$ -dependent vasodilation has been demonstrated in other vascular beds.^24^ Indeed, while there is contention regarding whether hemoglobin mediated RSNO,^7^ ${\mathrm{NO}}_{2}^{-}$ ,^25^ or ATP^26^ signaling are the primary hypoxic vasodilatory signal^1,23,27–30^ – particularly in *ex vivo* and animal models – our data in humans support the hemoglobin-based RSNO mechanism of vasodilation as a primary regulator of cerebral hypoxic vasodilation.

While the focus of our investigations was to assess the role of NO in regulating cerebral hypoxic vasodilation, the role of other prototypical vasodilators should not be disregarded. Previous studies conducted in humans have largely demonstrated that neither adenosine,^31^ prostaglandins,^32–34^ or reactive oxygen species,^35,36^ are obligatory for cerebral hypoxic vasodilation. However, Harrell and colleagues recently demonstrated that combined prostaglandin synthesis inhibition and ascorbic acid infusion results in a marked blunting of cerebral hypoxic vasodilation in humans (albeit with transcranial doppler blood velocity estimates of CBF), highlighting that ‘redundancy’ of vasodilatory pathways may underscore the lack of effect from vasodilator pathway blockades typically observed in human studies.^35^ Moreover, ATP sensitive potassium channels, which are influenced downstream of NO signaling by cyclic guanosine monophosphate, have been demonstrated to contribute to cerebral hypoxic vasodilation in humans.^37^ That ATP sensitive potassium channel blockade reduces cerebral hypoxic vasodilation further supports the role of NO observed in our study, although its effect may have manifested through the multiple other pathways that converge on ATP sensitive potassium channels.^38^ Therefore, while it is difficult to disentangle vasodilatory mechanisms in humans given the multiple levels of cross-talk that occur downstream of the ‘surface level’ pharmacological blockades that are safe to employ (e.g. adenosine receptor antagonism or prostaglandin synthesis inhibition),^38^ our results link physiologic changes in cerebral hypoxic vasodilation with alterations in NO bioavailability and trans-cerebral RSNO release in humans.

Following our observation that acute anemia (i.e. isovolumic hemodilution) augments the magnitude of cerebral hypoxic vasodilation for a given reduction in CaO_2_ (Figure 1(g)), and that this augmented cerebral hypoxic vasodilation was linked to greater trans-cerebral RSNO release (Figure 1(i) and (j)), we investigated this relationship further. Consistent across two separate investigations, we determined that a lower Hb concentration is associated with increased cerebral hypoxic vasodilation in both lowlanders (Figure 3(f)) and Andean highlanders (Figure 4(f)). Further, and consistent with investigation 1, an acute reduction in [Hb] (i.e. isovolumic hemodilution) led to an increase in cerebral hypoxic vasodilation in Andean highlanders with excessive erythrocytosis (Figure 4(o)). In both isovolumic hemodilution studies (investigation 1 & 4), the augmented cerebral hypoxic vasodilation may be explained by the greater level of hypoxia, which coincides with potentiated RSNO-mediated vasodilation^8,22^ as well as greater trans-cerebral RSNO release as observed herein (Figure 1(i)). Moreover, a lower [Hb] may be related to an improvement in NO-mediated signal transduction due to reduced NO scavenging by hemoglobin.^39–41^ However, we contend that it is unlikely that differences in blood viscosity associated with [Hb] underscore the differences in cerebral hypoxic vasodilation we observed (as reviewed in^42^).^43–46^

It is important to note that the relationship between Hb concentration and cerebral hypoxic vasodilation was only observed in males (Figure 3(f)). As investigations 1 and 2 were only conducted in males we are unable to discern if this is due to differences in the NO-dependence of cerebral hypoxic vasodilation between males or females or differing impacts of Hb levels on trans-cerebral RSNO release. Moreover, as menstrual phase was not standardized (or recorded) for investigation 3, it is unknown if the lack of relationship between cerebral hypoxic vasodilation is due to variability in female sex hormones (with potential impacts on NO signaling^47–49^). Nonetheless, this provides a more representative cross-section of female sex hormone profiles and thus a more generalizable sample of females than if inclusion was restricted to one menstrual phase (e.g. low circulating hormone phase as is common). Despite the lack of control over the menstrual phase, we observed a ∼20% greater cerebral hypoxic vasodilation in females when compared with males. As CaO_2_ will be lower for a given SaO_2_ in the females compared to males (due to lower [Hb]), the greater cerebral hypoxic vasodilation in females may be related to potentiated RSNO-mediated vasodilation.^8,22^

Given the low rate for which pre-clinical studies translate to the development of efficacious clinical therapies (<5%),^50,51^ demonstrating that an integral physiologic function is modifiable in humans, in this case by NO, is a key step in translation. Multiple cerebral hypoxic vasodilation signaling pathways have been elucidated in *ex vivo* and pre-clinical experiments (e.g. adenosine, prostaglandins) but when these studies are replicated (as much as one can) in humans, no effect is observed. If the manipulation of a signaling pathway is unable to modify an associated physiologic function in the context of human biology, then a treatment effect in clinical patients is unlikely. Our data provides a bridge between pre-clinical and clinical studies, whereby, in humans we demonstrate that cerebral hypoxic vasodilation is modifiable through changes in NO signaling. This sets a precedent for exploring NO-based therapies in disease populations where resolution of impairments in cerebral perfusion, acute or chronic, is desired (e.g. focal ischemic stroke or global hypoxic-ischemic brain injury).

## Conclusion

We determined that the magnitude of cerebral hypoxic vasodilation in humans is linked to trans-cerebral release of *S*-nitrosothiols. This implicates the erythrocyte as an integral regulator of cerebral blood flow. We further determined this vasodilation to be nitric oxide-dependent, observing a reduction in cerebral hypoxic vasodilation following nitric oxide synthase inhibition. Finally, we demonstrate during both acute experimental anemia, and when comparing across a cohort, that a lower hemoglobin concentration is related to increased cerebral hypoxic vasodilation. Collectively, these results indicate that hemoglobin and nitric oxide hold key roles in the regulation of cerebral hypoxic vasodilation in humans. That changes in nitric oxide-dependent signaling elicit changes in cerebral hypoxic vasodilation in humans indicates the potential feasibility of clinically modifying cerebral perfusion by manipulation of these pathways and thereby provides a promising therapeutic target for the treatment of brain diseases associated with acute or chronic impairments in cerebral perfusion.

## Supplemental Material

sj-pdf-1-jcb-10.1177_0271678X231169579 - Supplemental material for Hemoglobin and cerebral hypoxic vasodilation in humans: Evidence for nitric oxide-dependent and *S*-nitrosothiol mediated signal transduction Click here for additional data file. Supplemental material, sj-pdf-1-jcb-10.1177_0271678X231169579 for Hemoglobin and cerebral hypoxic vasodilation in humans: Evidence for nitric oxide-dependent and *S*-nitrosothiol mediated signal transduction by Ryan L Hoiland, David B MacLeod, Benjamin S Stacey, Hannah G Caldwell, Connor A Howe, Daniela Nowak-Flück, Jay MJR Carr, Michael M Tymko, Geoff B Coombs, Alexander Patrician, Joshua C Tremblay, Michelle Van Mierlo, Chris Gasho, Mike Stembridge, Mypinder S Sekhon, Damian M Bailey and Philip N Ainslie in Journal of Cerebral Blood Flow & Metabolism

## Data Availability

The data that support the findings of this study are available from the corresponding author upon reasonable request.
