# Supplementary material for: Hemoglobin and cerebral hypoxic vasodilation in humans: Evidence for nitric oxide-dependent and S-nitrosothiol mediated signal transduction
Source: J Cereb Blood Flow Metab. 2023 Apr 12;43(9):1519–31. doi: 10.1177/0271678X231169579 (PMC10414015; doi:10.1177/0271678X231169579)
Supplement: sj-pdf-1-jcb-10.1177_0271678X231169579 - Supplemental material for Hemoglobin and cerebral hypoxic vasodilation in humans: Evidence for nitric oxide-dependent and S-nitrosothiol mediated signal transduction [file sj-pdf-1-jcb-10.1177_0271678X231169579.pdf]

SUPPLEMENTAL MATERIAL

Hemoglobin and hypoxic cerebral vasodilation in humans: evidence for nitric oxide-dependent and *S*-nitrosothiol mediated signal transduction

Ryan L. Hoiland[1,2,3,4], David B. MacLeod[5], Benjamin S. Stacey[6], Hannah G. Caldwell[3] Connor A. Howe[3], Daniela Nowak-Flück[3], Jay M.J.R. Carr[3], Michael M. Tymko[3], Geoff B. Coombs[3], Alexander Patrician[3], Joshua C. Tremblay[3], Michelle Van Mierlo[7], Chris Gasho[8], Mike Stembridge[9], Mypinder S. Sekhon[4,10,11], Damian M. Bailey[6], Philip N. Ainslie[3].

[1]*Department of Anesthesiology, Pharmacology and Therapeutics, Vancouver General Hospital, West 12th Avenue, University of British Columbia, Vancouver, BC, Canada*
[2]*Department of Cellular and Physiological Sciences, Faculty of Medicine, University of British Columbia, Vancouver, BC, Canada*
[3]*Centre for Heart, Lung, and Vascular Health, School of Health and Exercise Sciences, Faculty of Health and Social Development, University of British Columbia Okanagan, Kelowna, BC, Canada*
[4]*International Collaboration on Repair Discoveries, West 10th Avenue, Vancouver, BC, Canada*
[5]*Human Pharmacology & Physiology Lab, Department of Anesthesiology, Duke University Medical Center, Durham, NC, 27708, USA*
[6]*Neurovascular Research Laboratory, Faculty of Life Sciences and Education, University of South Wales, Pontypridd, UK, CF37 4BB*
[7]*Department of Biomechanical Engineering, University of Twente, Enschede, The Netherlands*
[8]*Department of Medicine, Division of Pulmonary and Critical Care, Loma Linda University School of Medicine, Loma Linda, CA, USA*
[9]*Cardiff School of Sport and Health Sciences, Cardiff Metropolitan University, Cardiff, UK, CF23 6XD*
[10]*Djavad Mowafaghian Centre for Brain Health, Department of Pathology and Laboratory Medicine, Faculty of Medicine, University of British Columbia, Vancouver, BC, Canada*
[11]*Division of Critical Care Medicine, Department of Medicine, Vancouver General Hospital, West 12th Avenue, University of British Columbia, Vancouver, BC, Canada*

SUPPLEMENTAL MATERIAL: APPENDIX A – SUPPLEMENTAL METHODS

Supplemental Methods: Ethical Approval

All protocols described herein were approved by the University of British Columbia's Clinical Research Ethics Board (H16-01028; H18-01755; H18-01404; H18-01764), Duke University Institutional Review Board (Protocol: 00091879), the Universidad Peruana Cayetano Heredia Comité de Ética (no. 101686).[1] This study adhered to the standards outlined in Declaration of Helsinki (except registration in a database) and the Canadian Tri-council Policy Statement for Integrity in Research. Following a detailed description of study protocols and familiarization with the laboratory equipment and procedures written informed consent was provided in writing.

Supplemental Methods: Hemodilution and Nitric Oxide Inhibition Studies

For the Investigation 1 (isovolumic hemodilution) and Investigation 2 (nitric oxide synthase inhibition) the same general experimental measures were utilized for cardiovascular and respiratory monitoring. The protocols also shared the same cerebral blood flow monitoring techniques as well as arterial and jugular venous catheterization and blood sampling protocols. For the hemodilution study, participants attended the laboratory at 0600h (n=5) and 1200h (n=5) having fasted for greater than six hours as well as having abstained from exercise (24 hours), alcohol (24 hours), and caffeine (12 hours). Participants were further instructed to avoid foods high in nitrates for 48 hours prior to experimentation with specific instructions to avoid fruits, salads and cured meats.[2] For the nitric oxide synthase inhibition study, participants attended the laboratory at 0600h having fasted for greater than six hours, abstained from exercise (24 hours), alcohol (24 hours), and caffeine (12 hours). Participants were also instructed to avoid foods high in nitrates for 48 hours prior to experimentation.

Using sterile technique, a 20G arterial catheter (Arrow, Markham, ON) was advanced into the left radial artery under local anesthesia (Lidocaine, 1.0%). This technique was assisted via the use of ultrasound guidance. Subsequently, a 13G central venous catheter (Cook Medical, Bloomington, IN) was advanced into the right internal jugular vein, again under sterile conditions and with the use of local anesthesia and ultrasound guidance. The catheter was then advanced up to 15 cm cephalad.[3] The same technique, performed by the same clinician (DBM) has been previously demonstrated to lead to catheter tip placement in the jugular bulb, which is importantly proximal to the facial vein.[4] Further, correct placement was additionally determined by participants noting a sensation in their ear upon full insertion of the catheter.[3] Fulfilment of these techniques leads to ≤3% contamination by extra-cerebral blood.[3] Finally, an 18G venous catheter (Insyte™ Autoguard™, Becton Dickinson, USA) was inserted into the median ante-cubital vein. See **Supplemental Figure 1**.

Cardiovascular and respiratory variables were sampled continuously throughout the protocol at 1 KHz via an analog-to-digital converter (Powerlab, 16/30, ADInstruments, Colorado Springs, CO). Heart rate (HR) was measured by a lead-II configuration electrocardiogram (ADI bioamp ML132). Both the partial pressure of end-tidal CO_2 ($P_{ET}CO_2$) and O_2 ($P_{ET}O_2$) were sampled at the mouth and recorded by a calibrated gas analyzer (ML206, ADInstruments, Colorado, CO), while respiratory flow and minute ventilation (VE) were measured by a pneumotachograph (HR800L, HansRudolph, Shawnee, KS) connected in series with a bacteriological filter. The radial arterial and jugular venous catheters were both attached to an in-line and waste-less sampling system (VAMP system, Edwards Life Sciences). This allows for serial blood sampling and the

continuous measurement of radial arterial and jugular venous blood pressure (Truwave Transducer, Edwards Life Sciences). We calculated cerebral perfusion pressure (CPP) as the difference between mean arterial pressure (MAP) and jugular venous blood pressure (JBP), which is reflective of ICP in the pressure range of the current study [5].

$$\mathrm{CPP} = \mathrm{MAP} - \mathrm{JBP}$$

End-tidal forcing was utilized to control the partial pressure of end-tidal oxygen ($P_{ET}O_2$) for the purpose of targeting a specific partial pressure of arterial oxygen (PaO_2).[6,7] We predicted the necessary $P_{ET}O_2$ to target PaO_2 and subsequently achieve a desired arterial oxygen saturation (SaO_2) value by accounting for the known end-tidal-to-arterial gradient with our breathing apparatus [7] and the known relationship between PaO_2 and SaO_2 [8] (formalism presented below). Pilot testing confirmed the suitability of our $P_{ET}O_2$ targets for the study.

Prediction of PaO_2 from $P_{ET}O_2$ [7]:

$$PaO_2 = -6.024 + (0.986 \cdot P_{ET}O_2)$$
$$R^2 = 0.94;\ P < 0.001$$

Prediction of SaO_2 from estimated PaO_2 value [8]:

$$SaO_2 = (((PaO_2^3 + 150 \cdot PaO_2)^{-1} \cdot 23400) + 1)^{-1}$$

Blood velocity through the right MCA (MCAv) and left PCA (PCAv) was measured with a 2-MHz transcranial Doppler ultrasound (TCD) (Spencer Technologies, Seattle, WA). The TCD probes were attached to a specialized headband (Model M600 Bilateral Head Frame, Spencer Technologies) and secured in place. Insonation was achieved through the trans-temporal window superior to the zygomatic arch and rostral of the pinna by using previously described location and standardization techniques (these data are not reported).[9]

Blood velocity and vessel diameter of the ICA and VA were measured with a 10-MHz multi-frequency linear array duplex ultrasound (Terason uSmart 3300, Teratech, Burlington, MA). Arterial diameter was measured with B-mode imaging. Dynamic range (i.e., contrast) and gain (i.e., brightness) were consistent throughout the protocol within each vessel. Pulse-wave mode was used to simultaneously measure peak blood velocity. Insonation angle was constant (60 degrees) while sample volume was adjusted to span the entire intra-luminal diameter. Measures of ICA (ICA_{FLOW}) and VA (VA_{FLOW}) blood flow were made ipsilateral to the MCA and PCA, respectively. The ICA diameter and velocity were measured at least 1.5 cm distal to the common carotid bifurcation to eliminate recordings of turbulent and retrograde flow, while VA diameter and velocity were measured between C4–C5, C5–C6, or proximal to entry into the vertebral column. The location was determined on an individual basis in an attempt to select the most reproducible measures, with the same location repeated within participants and between trials.

Ultrasound recordings were screen captured and stored as video files for offline analysis. Concurrent outputs for arterial diameter and peak blood velocity were acquired at 30 Hz with customized edge detection and wall tracking software designed to mitigate observer bias [10]. One minute averages were used to determine ICA_{FLOW} and VA_{FLOW}. Volumetric cerebral blood flow (CBF) was calculated using the following formula:

$$\mathrm{ICA_{FLOW}\ or\ VA_{FLOW}} = \left(\frac{\text{Peak envelope velocity}}{2}\right) \cdot \pi \left(\frac{\text{diameter}}{2}\right)^2$$

$$CBF = 2 \cdot (ICA_{FLOW} + VA_{FLOW})$$

It is important to note that ~3% of the blood accounted for in this measurement of CBF is diverted from the brain via the ophthalmic artery.[11]

To account for MAP in our analysis of the CBF responses, cerebrovascular conductance (CVC) was subsequently calculated (e.g., CBF/CPP). We conducted ultrasound scanning in accordance with published technical recommendations[12] and have previously reported a within- and between-day coefficient of variation for extracranial artery scanning of 1.5 and 4.4%, respectively.[13]

At each stage of hypoxia ~1.0mL of radial arterial and jugular venous blood were simultaneously drawn into pre-heparinized syringes (SafePICO, Radiometer, Copenhagen, Denmark) and analyzed immediately using a commercial blood gas analyzer (ABL90 FLEX, Radiometer). This analysis included measurement of PaO_2, $PaCO_2$, SaO_2, CaO_2, pH, $[H^+]$, $[HCO_3^-]$, [Hb], Hct, and other parameters. Jugular venous blood was also analyzed for whole blood viscosity. Jugular venous blood was drawn into a Lithium Heparin Vacutainer® (Becton Dickinson, USA).

At specific experimental time points, arterial and jugular bulb blood samples were drawn into K_2EDTA Vacutainers® (Becton Dickinson, USA). Whole blood was then immediately centrifuged at 600 g for 10 minutes at a temperature of 4.0°C. Plasma was aliquoted into cryovials, flash frozen in liquid N_2, and stored at -80°C. Samples were later batch analyzed to measure plasma NO_2^- using tri-iodide reductive ozone based chemiluminescence, as previously described by our research group.[14–16] Samples were rapidly thawed at 37°C for 3-minutes, at which time 540 uL of plasma was mixed with 60 uL of 5% acidified sulphanilamide (9:1 ratio) and left to incubate in the dark at 21°C for 15-minutes to remove NO_2^- before injection into the tri-iodide reagent for the measurement of s-nitrosothiols (RSNO). Immediately following the mixing of plasma with acidified sulphanilamide (i.e., during the incubation period), two 100 uL plasma samples from the original cryovial were subsequently injected into the tri-iodide reagent for the combined measurement of NO_2^- and RSNO. These injections were separated by ≥2min to ensure recordings had reached a new stable baseline before subsequent injections. The tri-iodide reagent was then replaced prior to injection of the 400uL of the sample treated with acidified sulfanilamide. The average of the two 100uL injections was calculated. NO_2^- was calculated by subtracting the concentration of RSNO from the total NO_2^-+RSNO value. Our coefficient of variation for these measures is <10%. All calculations were performed using Origin 8 (OriginLab Corps, Massachusetts, USA) and smoothed using a 150-point averaging algorithm. The Peak Analysis package was used to calculate the area under the curve (mV), which was subsequently converted to a concentration, using standard curves of known concentrations measured using identical injection volumes. All chemicals were of the highest purity available from MilliporeSigma.

We combined our measures of the differing NO species with CBF to determine their net exchange as follows:

$$\text{Net exchange } (\text{nmol} \cdot \text{min}^{-1}) = \frac{CBF\ (mL \cdot min^{-1})}{1000} \cdot (1 - Hct) \cdot (a - vNO)$$

Supplemental Methods: Hemodilution Protocol

Following the first isocapnic hypoxemia test, the isovolumic hemodilution commenced. Whole blood was transferred into an Anticoagulant Citrate Phosphate Dextrose Solution BLOOD-PACK™ (4R0012MC, Fenwal, USA) through a non-pyrogenic plasma transfer kit (4C2240, Fenwal, USA) to collect up to 450mL of whole blood per BLOOD-PACK for short term storage. This blood was stored at room temperature on an orbital agitator.

The hemodilution protocol was conducted in two stages aimed at removing/replacing blood in 10% increments (i.e., 10% of whole blood volume). Thus approximately 10% of whole blood was removed and then replaced with an equal volume of 5% human serum albumin (Alburex 5%). Adequate hemodilution (±1% of target) was confirmed with an arterial [Hb] and Hct measurement. Subsequently, end-tidal forcing was utilized to return end-tidal gases to normal resting values in the event that any respiratory changes and end-tidal gas deviations had occurred during hemodilution. Here, a duplex ultrasound measures of CBF were conducted simultaneous to a radial arterial and jugular venous blood sample. The first stage of blood removal took 27±7 minutes, while replacement with albumin took 15±3 minutes. The second stage of blood removal took 26±6 minutes, while blood replacement with albumin took 14±5 minutes. These two stages of hemodilution were designed to match the hypoxemic stimulus at 90% and 75-80% SaO_2.

Supplemental Methods: Nitric Oxide Synthase Inhibition Protocol

Following instrumentation with a radial artery, jugular venous, and ante-cubital venous catheter (**Supplemental Figure 1**) participants had ≥20 minutes to rest while the remaining experimental set-up was completed. Thereafter, either a control (volume matched saline, 0.9% NaCl), nitric oxide synthase inhibitor (N^G-monomethyl-L-arginine, L-NMMA) or α_1-adrenoceptor agonist (phenylephrine) infusion was initiated. We utilized a singled-blinded counter-balanced experimental design where trials were separated by 5 h, the equivalent of five half-lives for l-NMMA.[17] The L-NMMA infusion was initiated with a 5 mg/kg bolus delivered over 5 min followed by a 50µg/kg/min maintenance dose throughout the protocol until completion. Further, in a subset of five of the same participants, we also completed a phenylephrine infusion (0.1–0.6µg/kg/min constant infusion;[18]). Three participants received phenylephrine as their first infusion, and two as their second. The phenylephrine infusion was conducted as an equipotent vasoconstrictor control for the L-NMMA trial[18]

During each drug infusion we conducted an isocapnic hypoxemia protocol with a baseline period followed by two hypoxic stages lasting 5-minutes each where SaO_2 was targeted to 85% and 75%. At baseline and each stage of hypoxia, $\dot{V}_E$, $P_{ET}CO_2$, $P_{ET}O_2$, HR, MAP, JVBP, MCA, PCA, VA, ICA, and blood gas measures were made.

Supplemental Methods: Baseline hemoglobin concentration and hypoxic cerebral vasodilation protocol

Data were analyzed from a repository made available through a collaboration with Duke University (Durham, NC, USA). The data repository analyzed for this study consisted of nine separate experiments conducted between 2004 and 2016 with a total of 199 study participants. A breakdown of the studies included is presented in **Supplemental Table 1**. Each study consisted of a graded isocapnic hypoxemia test to a final SaO_2 of 70%. However, as the data are derived from separate protocols, the number of stages was variable and ranged from four to seven stages. End-tidal gas control during the hypoxemia tests was conducted using the RespirAct™ (Thornhill Medical, Toronto) [19] or end-tidal forcing.[6]

Subjects underwent the previously described radial arterial and jugular venous cannulation (**Supplemental Figure 1**). This blood was analyzed as previously described with an ABL90 FLEX for PaO_2, $PaCO_2$, SaO_2, CaO_2, pH, [H^+], [HCO_3^-], [Hb], Hct. Trans-cerebral blood draws were acquired serially at each stage of hypoxemia. In these studies there were no ultrasound measurements conducted. Thus, percentage changes in CBF at each stage of hypoxemia were determined based upon the Fick Principle. That is, the cerebral metabolic rate

of oxygen ($CMRO_2$) is the product of CBF and the arterio-venous difference of oxygen ($AvDO_2$). Therefore, CBF is the quotient of $CMRO_2$ and the $AvDO_2$. As $CMRO_2$ is constant in hypoxia (see **Figure 3** in the manuscript) [4], CBF is inversely proportional to $AvDO_2$ and percent reductions in $AvDO_2$ with hypoxemia indicate percent increases in CBF (see **Figure 3** in the manuscript). The formalism guiding these calculations is provided below:

$$CMRO_2 = CBF \cdot avDO_2 \qquad \text{therefore,}$$

$$CBF = \frac{CMRO_2}{avDO_2} \qquad \text{therefore,}$$

Under the assumption of a constant $CMRO_2$

$$CBF \propto avDO_2^{-1}$$

As noted, the number of hypoxemic stages was variable; however, given the CBF response in this range of hypoxemia is linear [20], hypoxic CBF reactivity slopes are comparable across all experiments included. Therefore, hypoxemic cerebrovascular reactivity (i.e. magnitude of hypoxic cerebral vasodilation) was calculated as the slope of the linear regression between percent changes in CBF and absolute reductions in CaO_2 [i.e., %ΔCBF · ΔCaO_2 (mL/dL)]. Prior to further data and statistical analyses, this data repository was scrutinized for data quality control purposes. The following data exclusion criteria were used:

1) Data were excluded if one or more of the primary parameters for analysis (CaO_2, $CjvO_2$, & [Hb]) were missing (n=37);
2) If the participant terminated the isocapnic protocol prior to reaching 70% SaO_2 (n=5);
3) If the relationship between CBF and CaO_2 was not linear (R^2 <0.7 arbitrary cutoff; n=6);
4) If there was a $PaCO_2$ deviation of ≥3mmHg during any stage, which would largely confound the %change in CBF (n=17).

This resulted in the exclusion of 65 participants and a final sample size of 134 (79 male, 55 female).

Supplemental Methods: Peru Study

High-altitude native Andeans with (n=15; all male; Age: 39±15 years; body mass index: 25±3 kg/m^2) and without (n=25; 2 female; Age: 29±12 years; body mass index: 24±3 kg/m^2) excessive erythrocytosis (EE+ / EE-) were recruited to participate in the Cerro de Pasco region of Peru (4,300 m above sea-level). Upon initial enrollment a venous blood sample was collected for the measurement of Hct and [Hb]. Duplex ultrasound measures of VA and ICA flow, peripheral oxyhemoglobin saturation (SpO_2, pulse oximetry), and resting blood pressure were made (automated brachial blood pressure cuff). Male participants were defined as EE+ with a [Hb] ≥21 g/dL while female participants were defined as EE+ with a [Hb] ≥19 g/dL.[21] To estimate CaO_2 in these participants, we used the following equation:

$$\mathrm{CaO_2(mL/dL)} = (1.34 \cdot [\mathrm{Hb}] \cdot (\mathrm{SpO_2}/100))$$

Thereafter, 15 participants (EE-, n=9; EE+, n=6) participated in a substudy assessing the CBF response to isocapnic hypoxemia. An isocapnic hypoxemia test was conducted, wherein $P_{ET}O_2$ was targeted to 100 mmHg to restore SaO_2 to normal (i.e. 98%), and then reduced to a

$P_{ET}O_2$ of 50 and 40 mmHg, with each stage lasting 5-minutes following the attainment of steady-state. At each stage, $\dot{V}_E$, $P_{ET}CO_2$, $P_{ET}O_2$, HR, MAP, SpO_2, VA, and ICA measures were made.

In the six EE+ participants, this isocapnic hypoxemia test was conducted as part of a larger hemodilution protocol. Thus, following the initial isocapnic hypoxemia test, six EE+ participants completed an isovolumic hemodilution protocol (Age: 44±19 years; body mass index: 25±3 kg/m^2). Therein, 25% of blood volume was removed and replaced with human serum albumin (5%), The removal and replacement was conducted simultaneously and in 1-stage until completion. Following hemodilution these participants underwent a follow up isocapnic hypoxemia test, wherein $P_{ET}O_2$ was targeted to 100 mmHg to restore SaO_2 to normal (i.e. 98%), and then reduced to a $P_{ET}O_2$ of 50 and 40 mmHg, with each stage lasting 5-minutes following the attainment of steady-state. At each stage, $\dot{V}_E$, $P_{ET}CO_2$, $P_{ET}O_2$, HR, MAP, VA, and ICA measures were made. Further, these six participants were instrumented with a radial artery catheter so that blood gases could be collected at each baseline and stage of hypoxemia (thus CaO_2 was not estimated, but rather quantified by arterial blood gas analyses in these six participant).

Supplemental Methods: Statistical Analyses

For all statistical tests, significance was set at an alpha level of α=0.05. Data were assessed for normality using Shapiro-Wilks W tests. Normal data are reported as mean±standard deviation. When data significantly differed from a normal distribution, non-parametric analyses were utilized and data are were presented as median [interquartile range].

Investigation 1. Differences in variables at baseline prior to and following hemodilution were compared with two-tailed paired t-tests. Changes in CBF and CDO_2 during the initial hypoxemia trial, hemodilution, and the post-hemodilution hypoxemia trial were analyzed using linear mixed effects modelling. Fixed effects were the individual stages and the experimental trial (hypoxemia pre, hemodilution, hypoxemia post), with subject included as a random effect. When significant effects were detected, post-hoc comparisons were conducted using a Bonferroni correction. To compare the slope of the relationship between gCBF / CDO_2 and CaO_2 across each trial (i.e. cerebral hypoxic vasodilation), we used linear mixed effects modelling.[22] Fixed effects were CaO_2 and the experimental trial (hypoxemia pre, hemodilution, hypoxemia post), with subject included as a random effect. This model was tested against the same model, but with CPP included as a co-variate. Inclusion of CPP significantly improved the model. Thus, CPP was accounted for as a co-variate in our analyses of CBF reactivity. Residuals were examined for parity to normality. Changes in the a-v differences and cerebral net exchange of NO_2^- and RSNOs were analyzed with a 1-way repeated measures analysis of variance (ANOVA). Finally, the relationship between the a-v difference of NO_2^- and RSNOs with gCBF was assessed with repeated measures correlations.[23]

Investigation 2. Plasma NO levels during saline and L-NMMA infusion were compared with a two-tailed paired samples t-test. The CPP, CBF, CDO_2, and CVC responses to hypoxemia during saline and L-NMMA infusions were assessed with two-way repeated measures ANOVAs. When significant effects were detected, post-hoc comparisons were conducted using a Bonferroni correction. The slope of the relationship between CBF / CDO_2 / CVC with CaO_2 was determined using linear regression and compared between saline and L-NMMA infusion with two-tailed paired t-tests. Comparisons between the saline and phenylephrine trials followed the same statistical

models. Hypoxic reactivity slopes were compared between the saline and L-NMMA infusion trials using two-tailed paired t-tests or Wilcoxin Signed ranks tests where appropriate.

Investigation 3. The data repository consisted of 199 participants. It was assessed for missing data, prematurely terminated protocols or technical difficulties, which led to the exclusion of 65 participants (see **Supplemental Methods**) and a final sample size of 134 (79 male, 55 female). The relationship between resting [Hb] and hypoxic reactivity was assessed using Pearson r correlations. Males and females were also analyzed separately. Further, baseline variables and reactivity were compared between males and females using unpaired t-tests.

Investigation 4. Baseline characteristics were compared between EE- and EE+ participants with two-tailed unpaired t-tests. The relationships between CaO_2 and CBF as well as arterial [Hb] and CBF reactivity were assessed using Pearson r correlations. In the isovolumic hemodilution protocol, pre-post hemodilution comparisons were made using two-tailed paired t-tests. Changes in CBF and CDO_2 with hypoxemia prior to and following hemodilution were assessed with two-way repeated measures ANOVAs. When significant effects were detected, post-hoc comparisons were conducted using a bonferroni correction. The slope of the relationship between CBF and CDO_2 with CaO_2 was determined using linear regression and compared between pre- and post-hemodilution with two-tailed paired t-tests. The association between changes in CBF and CDO_2 reactivity and baseline arterial [Hb] were assessed with Pearson r correlations.

SUPPLEMENTAL FIGURES

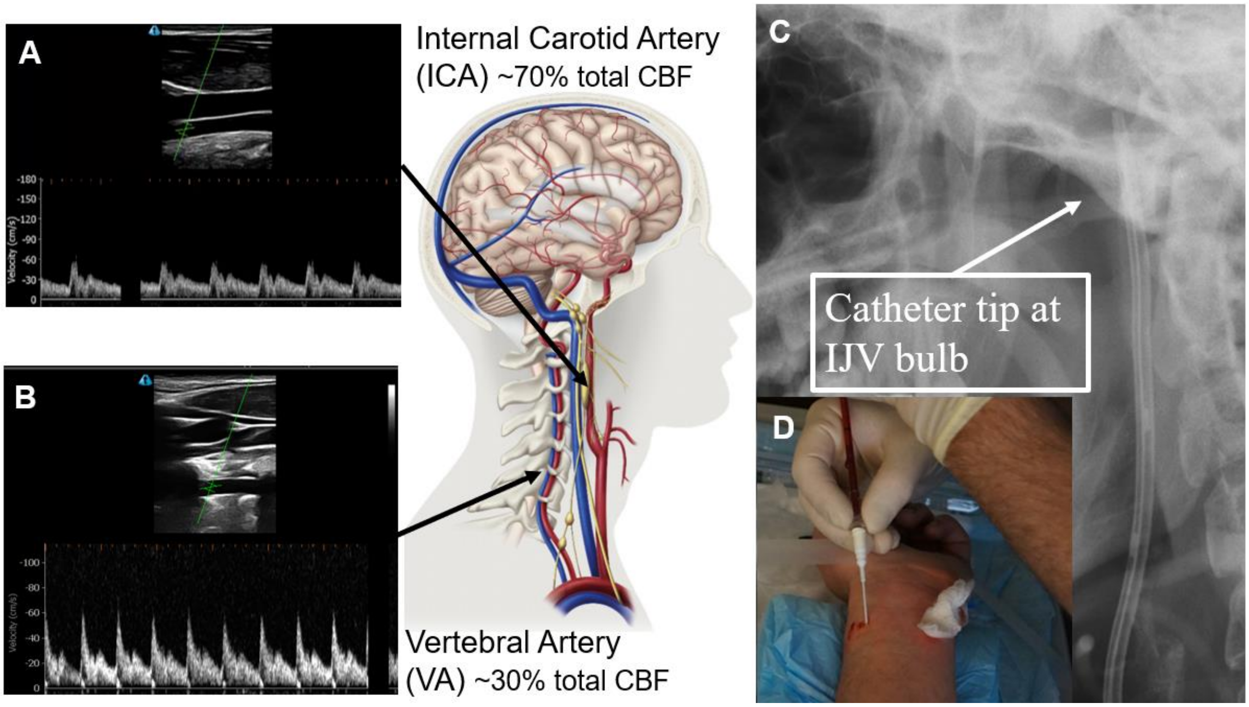

Supplemental Figure 1. Experimental model for measuring cerebral exchange kinetics. Panel A and B depict duplex ultrasound images of the internal carotid artery (ICA) and vertebral artery (VA) respectively, the measurements of which were utilized to calculate cerebral blood flow (CBF = 2 x (ICA_{FLOW} + VA_{FLOW}). Panel C is an X-ray of a participant demonstrating the internal jugular venous (IJV) bulb catheter tip placement, while Panel D depicts insertion of a radial arterial line for the acquisition of arterial blood samples. Collectively, measures of cerebral blood flow and paired sampling of arterial and jugular venous blood are utilized to calculate trans-cerebral exchange kinetics (see calculations in supplemental methods).

SUPPLEMENTAL TABLES

Supplemental Table 1. Individual studies to assess the relationship between hemoglobin concentration and hypoxic cerebral vasodilation in Investigation 3.

Study	Year	n, subjects	n, subjects included in final analysis	n, stages of hypoxemia
1	2004	21	14	5
2	2007	19	18	7
3	2009	23	19	7
4	2011	9	8	4
5	2011	42	33	7
6	2011	9	8	7
7	2013	26	23	6
8	2014	35	28	6
9	2016	15	15	4

Supplemental Table 2. Participant data for Investigation 3.

**Variable**	**All Subjects**	n	**Male**	n	**Female**	n	**P-value**
Subjects, n	134		79		55		
Age, years	26± 6	(117)	26 ± 6	(69)	25 ± 4	(55)	0.10
Height, cm	173 ± 9	(116)	179 ± 6	(68)	165 ± 7	(55)	**< 0.01**
Weight, kg	72 ± 13	(116)	79± 11	(68)	62±8	(55)	**< 0.01**
BMI, kg/m^2	24± 3	(116)	25 ± 3	(68)	23 ± 3	(55)	**< 0.01**
MAP, mmHg	86.6 ± 9.3	(86)	89.2±9.4	(36)	83.7 ±8.2	(30)	<**0.01**
[Hb], g/dL (arterial)	14.1 ± 1.3	(134)	14.8 ± 1.0	(79)	13.1 ± 1.2	(55)	**< 0.01**
HCT, % (arterial)	41.7 ± 3.8	(120)	43.6 ± 3.2	(67)	39.3 ± 3.1	(55)	**< 0.01**
$\%\Delta CBF \cdot \Delta CaO_2$	-7.86±3.6	(134)	-7.54±4.13	(79)	-8.32±2.71	(55)	**<0.01**

Data are presented as mean ± standard deviation. The P-value represents the statistical output for a t-test between men and women.

SUPPLEMENTAL REFERENCES

1. Tymko MM, Stembridge M, Patrician A, Moore JP, Meah VL, Macleod DB, Bain AR, Hoiland RL, Dawkins TG, Howe CA, Simpson LL, Stacey BS, Gasho C, Lawley JS, Tremblay JC, Coombs GB, Gibbons TD, Steinback CD, Bailey DM, Anholm JD, Villafuerte FC, Vizcardo-galindo G, Ainslie PN. The 2018 Global Research Expedition on Altitude Related Chronic Health (Global REACH) to Cerro de Pasco, Peru: an Experimental Overview. *Exp Physiol*. 2021;106:86–103.
2. Wang J, Brown MA, Tam PSH, Chan MC, Whitworth JA. Effects of diet on measurement of nitric oxide. *Clin Exp Pharmacol Physiol*. 1997;418–420.
3. Schell RM, Cole DJ. Cerebral monitoring: jugular venous oximetry. *Anesth Analg*. 2000;90:559–566.
4. Ainslie PN, Shaw AD, Smith KJ, Willie CK, Ikeda K, Graham J, Macleod DB. Stability of cerebral metabolism and substrate availability in humans during hypoxia and hyperoxia. *Clin Sci (Lond)*. 2014;126:661–70.
5. Cardim D, Griesdale DE, Ainslie PN, Robba C, Calviello L, Czosnyka M, Smielewski P, Sekhon MS. A comparison of non-invasive versus invasive measures of intracranial pressure in hypoxic ischaemic brain injury after cardiac arrest. *Resuscitation* [Internet]. 2019;137:221–228. Available from: https://doi.org/10.1016/j.resuscitation.2019.01.002
6. Tymko MM, Hoiland RL, Kuca T, Boulet LM, Tremblay JC, Pinske BK, Williams AM, Foster GE. Measuring the human ventilatory and cerebral blood flow response to CO2: A technical consideration for the end-tidal-to-arterial gas gradient. *J Appl Physiol*. 2016;120:282–296.
7. Tymko MM, Ainslie PN, Macleod DB, Willie CK, Foster GE. End-tidal-to-arterial CO2 and O2 gas gradients at low- and high-altitude during dynamic end-tidal forcing. *Am J Physiol Regul Integr Comp Physiol*. 2015;308:R895-906.
8. Severinghaus J. Simple, accurate equations for human blood O2 dissociation computations. *J Appl Physiol*. 1979;46:599–602.
9. Willie CK, Colino FL, Bailey DM, Tzeng YC, Binsted G, Jones LW, Haykowsky MJ, Bellapart J, Ogoh S, Smith KJ, Smirl JD, Day TA, Lucas SJ, Eller LK, Ainslie PN. Utility of transcranial Doppler ultrasound for the integrative assessment of cerebrovascular function. *J Neurosci Methods*. 2011;196:221–237.
10. Woodman RJ, Playford DA, Watts GF, Cheetham C, Reed C, Taylor RR, Puddey IB, Beilin LJ, Burke V, Mori T a, Green D. Improved analysis of brachial artery ultrasound using a novel edge-detection software system. *J Appl Physiol (1985)*. 2001;91:929–937.
11. Zarrinkoob L, Ambarki K, Wåhlin A, Birgander R, Eklund A, Malm J. Blood flow distribution in cerebral arteries. *Journal of Cerebral Blood Flow & Metabolism*. 2015;35:648–654.
12. Thomas KN, Lewis NCS, Hill BG, Ainslie PN. Technical recommendations for the use of carotid duplex ultrasound for the assessment of extracranial blood flow. *Am J Physiol Regul Integr Comp Physiol*. 2015;309:R707–R720.
13. Hoiland RL, Tymko MM, Bain AR, Wildfong KW, Monteleone B, Ainslie PN. Carbon dioxide mediated vasomotion of extra-cranial cerebral arteries in humans: A role for prostaglandins? *Journal of Physiology*. 2016;594:3463–3481.
14. Bailey DM, Rasmussen P, Overgaard M, Evans KA, Bohm AM, Seifert T, Brassard P, Zaar M, Nielsen HB, Raven PB, Secher NH. Nitrite and S-Nitrosohemoglobin Exchange Across the Human Cerebral and Femoral Circulation: Relationship to Basal and Exercise Blood Flow Responses to Hypoxia. *Circulation*. 2017;135:166–176.

15. Hoiland RL, Tremblay JC, Stacey BS, Coombs GB, Tymko MM, Patrician A, Stembridge M, Connor A, Bailey DM, Green DJ, Macleod DB, Ainslie PN. Acute reductions in hematocrit increase flow-mediated dilation independent of resting nitric oxide bioavailability in humans. *Journal of Physiology*. 2020;1–34.
16. Hoiland RL, Caldwell HG, Carr JMJR, Howe CA, Stacey BS, Dawkins T, Wakeham DJ, Tremblay JC, Tymko MM, Patrician A, Smith KJ, Sekhon MS, MacLeod DB, Green DJ, Bailey DM, Ainslie PN. Nitric oxide contributes to cerebrovascular shear-mediated dilation but not steady-state cerebrovascular reactivity to carbon dioxide. *Journal of Physiology*. 2021;
17. Mayer BX, Mensik C, Krishnaswami S, Derendorf H, Eichler HG, Schmetterer L, Wolzt M. Pharmacokinetic-pharmacodynamic profile of systemic nitric oxide-synthase inhibition with L-NMMA in humans. *Br J Clin Pharmacol*. 1999;47:539–544.
18. Ide K, Worthley M, Anderson T, Poulin MJ. Effects of the nitric oxide synthase inhibitor L-NMMA on cerebrovascular and cardiovascular responses to hypoxia and hypercapnia in humans. *J Physiol*. 2007;584:321–332.
19. Slessarev M, Han J, Mardimae A, Prisman E, Preiss D, Volgyesi G, Ansel C, Duffin J, Fisher J a. Prospective targeting and control of end-tidal CO2 and O2 concentrations. *J Physiol*. 2007;581:1207–19.
20. Hoiland RL, Bain AR, Tymko MM, Rieger MG, Howe CA, Willie CK, Hansen AB, Flück D, Wildfong KW, Stembridge M, Subedi P, Anholm JD, Ainslie PN. Adenosine receptor-dependent signaling is not obligatory for normobaric and hypobaric hypoxia-induced cerebral vasodilation in humans. *J Appl Physiol*. 2017;122:795–808.
21. Villafuerte FC, Corante N. Chronic Mountain Sickness: Clinical Aspects, Etiology, Management, and Treatment. *High Alt Med Biol*. 2016;17:61–69.
22. Atkinson G, Batterham AM, Jones H, Taylor CE, Willie CK, Tzeng YC. Appropriate within-subjects statistical models for the analysis of baroreflex sensitivity. *Clin Physiol Funct Imaging*. 2011;31:80–82.
23. Bakdash JZ, Marusich LR. Repeated measures correlation. *Front Psychol*. 2017;8:456.
